# Electronic non-adiabatic states: towards a density functional theory beyond the Born–Oppenheimer approximation

**DOI:** 10.1098/rsta.2013.0059

**Published:** 2014-03-13

**Authors:** Nikitas I. Gidopoulos, E. K. U. Gross

**Affiliations:** 1 Department of Physics, Durham University, South Road, Durham DH1 3LE, UK; 2 Max Planck Institute of Microstructure Physics, Weinberg 2, 06120 Halle (Saale), Germany

**Keywords:** beyond Born–Oppenheimer, density functional theory, non-adiabatic effects

## Abstract

A novel treatment of non-adiabatic couplings is proposed. The derivation is based on a theorem by Hunter stating that the wave function of the complete system of electrons and nuclei can be written, without approximation, as a Born–Oppenheimer (BO)-type product of a nuclear wave function, *X*(*R*), and an electronic one, *Φ*_*R*_(*r*), which depends parametrically on the nuclear configuration *R*. From the variational principle, we deduce formally exact equations for *Φ*_*R*_(*r*) and *X*(*R*). The algebraic structure of the exact nuclear equation coincides with the corresponding one in the adiabatic approximation. The electronic equation, however, contains terms not appearing in the adiabatic case, which couple the electronic and the nuclear wave functions and account for the electron–nuclear correlation beyond the BO level. It is proposed that these terms can be incorporated using an optimized local effective potential.

## 1. Introduction

The Born–Oppenheimer (BO) [1,2] or adiabatic [3] separation of the electronic and nuclear motion forms the basis of almost any quantum mechanical study of molecules and solids. The method is based on the large difference between the electronic and nuclear masses, which implies that the light electrons are able to adapt almost instantaneously to the configuration of the much heavier nuclei. Consider a molecule with *N*_e_ electrons and *N* nuclei with masses *M*_*α*_ and charges *Z*_*α*_ and denote by *r* the set of electronic coordinates *r*=**r**_1_…**r**_*N*_e__ and by *R* the set of nuclear coordinates *R*=**R**_1_,…,**R**_*N*_.

The separation is effected by decomposing the complete molecular wave function, *Ψ*_mol_(*r*,*R*), in terms of a product of an electronic wave function depending parametrically on the nuclear positions and a nuclear wave function:
1.1
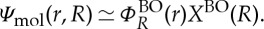


As the electrons, more or less, cannot see the nuclear motion, the electronic wave function is chosen to be an eigenfunction of an electronic Hamiltonian derived from the molecular one by ignoring the nuclear kinetic energy or, equivalently, by setting the nuclear mass to infinity. Finally, following Longuet-Higgins and Messiah, the equation for the nuclear wave function can be obtained variationally [3,4]: keeping the electronic wave function fixed, one requires that the expectation value of the molecular Hamiltonian in terms of the adiabatic product wave function remains stationary when the nuclear wave function is varied. In the resulting effective nuclear Hamiltonian, in the absence of electronic degeneracies, the nuclei lie in a potential which, to high accuracy, coincides with the electronic energy eigenvalue. Each electronic level allows for a set of nuclear vibrational and rotational excitations but does not couple directly to any nuclear state of this set. For many physical systems and phenomena, this separation is extremely successful, at least whenever degeneracy or near degeneracy of the electronic Hamiltonian is not involved. There are however other phenomena, which crucially depend on the coupling between the electronic and the nuclear wave functions. For these phenomena, the adiabatic or BO approximation breaks down.

One should be careful with the variational derivation of the nuclear BO equation, because the ground state of a free molecule is a continuum state even after separating away the centre of mass motion [5]. In principle, to use the variational principle, one should first separate off the overall translations [6] and the rotations of the molecule and then perform the factorization of the resulting bound wave function. This leads to complicated terms in the resulting Hamiltonian [7–11]. In this paper, we deliberately do not separate off translations and rotations, because our main aim is to show how one may improve upon the BO scheme, keeping at the same time the formulae simple and transparent. One may justify the use of the variational principle by imposing a confining potential that binds the molecular system within a very large but finite volume. In addition, we are interested not only in the study of molecules but also of crystalline solids, where periodic boundary conditions remove the overall translational and rotational motions of the system.

In probability theory, the joint probability distribution *p*(*r*,*R*) of two sets of variables *r*,*R* can be factorized as the product *p*(*r*,*R*)=*p*(*r*|*R*)*p*(*R*) of a conditional probability distribution *p*(*r*|*R*) for the (electronic) variables *r* given *R*, times a marginal probability distribution *p*(*R*) for the (nuclear) variables *R*. Hunter proposed accordingly that the molecular wave function *Ψ*_mol_(*r*,*R*), i.e. the joint probability density amplitude of electronic and nuclear degrees of freedom, could also be decomposed exactly as a product of a conditional probability amplitude *Φ*_*R*_(*r*) of electronic variables, given fixed values for the nuclear variables times a marginal probability amplitude *X*(*R*) for the nuclear degrees of freedom [12]:
1.2
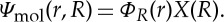
The BO electronic and nuclear wave functions 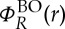, *X*^BO^(*R*) provide excellent approximations for *Φ*_*R*_(*r*) and *X*(*R*) but Hunter suggested that for each *Ψ*_mol_(*r*,*R*) one may find a conditional and a marginal probability density amplitude, such that the factorization is exact. Czub and Wolniewicz discovered that for diatomic molecules, *X*(*R*) is a nodeless wave function, and hence the potential in the Schrödinger equation determining *X*(*R*) must necessarily demonstrate spikes at the points were the adiabatic wave function has nodes [13]. We shall refer to the latter potential as the nuclear non-adiabatic potential (see 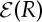 in equation (2.10)).

Hunter observed that this result is more general [14,15]. The proof is straightforward: take a molecular wave function that is to a good approximation given by a single BO product, 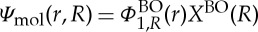, with the electronic part in the ground state and the nuclear wave function in an excited state with a node at *R*_0_. As the electronic adiabatic states form a complete set, the molecular wave function can be expanded exactly in the series
1.3
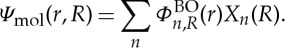
Consequently, the diagonal of the nuclear *N*-body density matrix |*X*(*R*)|^2^ is equal to
1.4
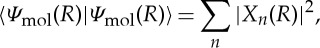
where 〈*Ψ*_mol_(*R*)|*Ψ*_mol_(*R*)〉 is the diagonal of the reduced density matrix of *Ψ*_mol_(*R*) in which the electronic degrees of freedom have been integrated out.

Here and in the following, integration over all electronic degrees of freedom is denoted by the bracket symbols, while integration over the nuclear degrees of freedom is written as 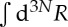.

In order for 〈*Ψ*_mol_(*R*)|*Ψ*_mol_(*R*)〉 to have a node at *R*_0_, all vibrational states *X*_*n*_(*R*) must have a node at the same *R*_0_, which cannot happen. As an interesting aside, we note that Hunter's argument fails for nodes dictated by symmetry. For example, for identical fermionic nuclei, the molecular wave function must be antisymmetric with respect to exchange of two nuclei, say at positions **R**_1_, **R**_2_ and then the diagonal *N*-body nuclear density matrix, and consequently the marginal probability amplitude must have nodes at (**R**_1_,**R**_2_)=(**R**,**R**).

So far, the impact of the work on the exact factorization of the molecular wave function has been limited and these ideas have not led to new practical ways to improve upon the adiabatic approximation. Perhaps in the early papers, attention focused too heavily on the nodeless character of the marginal probability density amplitude, which admittedly is a non-adiabatic coupling but probably not a very important one, because when the BO approximation is accurate, the correction does not have a measurably significant effect and when the BO approximation breaks down, it is not owing to the nodeless property of the wave function. More importantly, the non-adiabatic equation determining the electronic wave function *Φ*_*R*_(*r*), to the best of our knowledge, was never given by Hunter. Knowledge of that equation would inform us of the non-adiabatic terms that correct the BO electronic wave function and hopefully accurate approximate treatments of these terms would emerge. This is the goal of our paper.

There is a representability issue which perhaps was not addressed adequately: namely it is not clear whether the marginal probability distribution always corresponds to a meaningful nuclear wave function. For example, the decomposition that was proposed to demonstrate the factorization (1.2) with 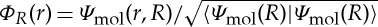 and 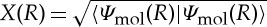 implies that the nuclear wave function is always bosonic even when the nuclei are fermionic. This problem can be overcome by introducing an *R*-dependent phase and writing
1.5
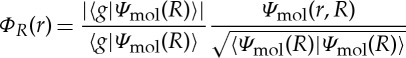
and
1.6
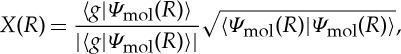
where *g*(*r*) is a suitably chosen electronic wave function that does not depend on *R*. In this way, the electronic wave function *Φ*_*R*_(*r*) (conditional probability amplitude) depends on *R* and for identical nuclei is symmetric with respect to nuclear exchange. The nuclear wave function *X*(*R*) (marginal probability amplitude) has the same symmetry properties as *Ψ*_mol_(*r*,*R*) with respect to nuclear exchange.

Adopting the small potential mentioned earlier to confine the molecule in a very large volume, the molecular, electronic and nuclear wave functions are all normalized to unity for convenience
1.7
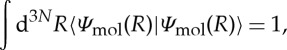

1.8
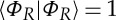

and
1.9
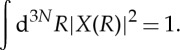


The electronic and nuclear wave functions have the freedom of an *R*-dependent phase (which, for identical nuclei must be symmetric with respect to exchange)
1.10
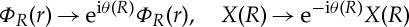
because this transformation leaves *Ψ*_mol_(*r*,*R*) invariant. Apart from this freedom of phase the components *X* and *Φ*_*R*_ are unique. To prove this uniqueness, we consider two representations, *Φ*_*R*_⋅*X* and 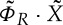, of the exact molecular wave function, i.e.
1.11
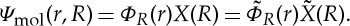
This implies that the ratio of 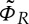 and *Φ*_*R*_ depends on *R* only
1.12
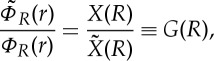
where, from now on, the ratio of *X*(*R*) and 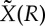 will be denoted by *G*(*R*). Hence,
1.13
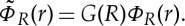
Using normalization condition (1.8) for *Φ*_*R*_ and 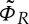, equation (1.13) leads to
1.14
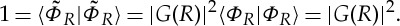
Hence, we conclude
1.15
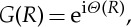
which proves the uniqueness of *Φ*_*R*_ and *X* up to within an *R*-dependent phase:
1.16
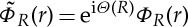
and
1.17
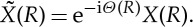
Note that it is condition (1.8) that requires *Φ*_*R*_(*r*) to be normalized to 1 for each separate nuclear configuration *R*, which leads to the uniqueness (within a phase) of *Φ*_*R*_ and *X*.

## 2. Non-adiabatic electronic and nuclear equations

As the parametric product ansatz (1.2) does not constitute an approximation, it should be possible to improve significantly upon the BO level of accuracy, still using a product wave function, if we optimize the electronic wave function in equation (1.2) variationally, under normalization constraint (1.8), rather than choose it *a priori* to be an eigenstate of the BO electronic Hamiltonian. Naturally, we expect that in the non-adiabatic scheme, the electronic wave function will be coupled to the nuclear state.

The molecular Hamiltonian has the form
2.1
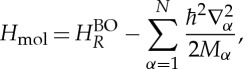
where 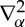 is the Laplacian with respect to **R**_*α*_ and *H*^BO^_*R*_ is the electronic Hamiltonian
2.2
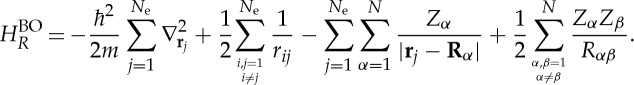
The nuclear Coulomb energy (last term) is independent of *r* and represents a constant shift of the electronic energy.

In order to obtain the equations determining the electronic and the nuclear wave functions, *Φ*_*R*_(*r*) and *X*(*R*), we have to vary the expectation value of the Hamiltonian *H*_mol_ with respect to *Φ*_*R*_(*r*) and *X*(*R*), under normalization constraints (1.8) and (1.9). The latter are incorporated through the use of Lagrangian multipliers *Λ*′(*R*), *E*. Note that we have infinitely many constraints for the normalization of the electronic wave function, one for each *R*, and we need as many Lagrangian multipliers *Λ*′(*R*):
2.3
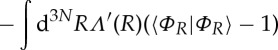
and
2.4
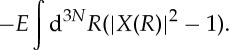
A few words about notation—if the gradient operator ∇_*α*_, or the Laplacian 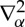 lies within parentheses or between brackets, as in 〈*Φ*_*R*_|∇_*α*_*Φ*_*R*_〉, it is implied that it acts only inside the brackets or parentheses. Note that when we use *square brackets*, as in 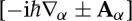, the action of ∇_*α*_ is not confined within the brackets. Direct optimization of the electronic and nuclear wave functions under constraints (2.3) and (2.4) yields the non-adiabatic equations
2.5
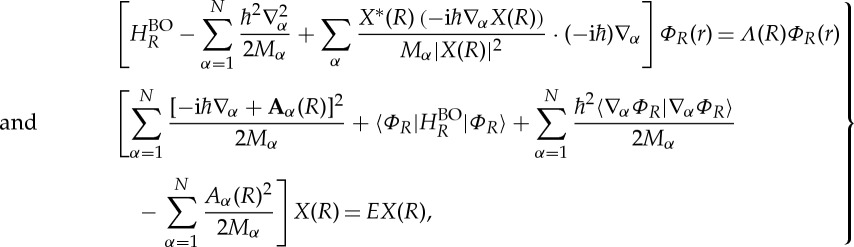
where *Λ*(*R*)=*Λ*′(*R*)/|*X*(*R*)|^2^ and **A**_*α*_(*R*) is the vector-potential-like real quantity
2.6
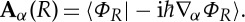
To make further progress, we define an electronic energy as
2.7
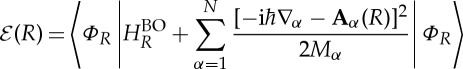
which, by equation (2.6), can be written as
2.8
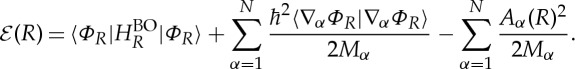
After some straightforward algebra, equations (2.5) and (2.6) can be expressed in terms of this electronic energy as
2.9
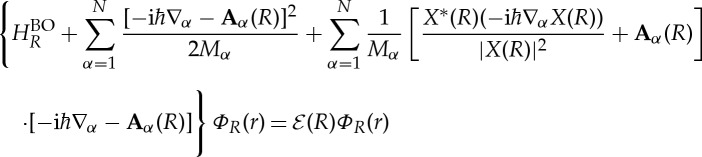
and
2.10
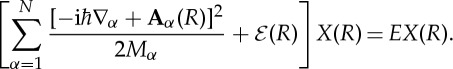


While, from the numerical point of view, this form of the equations is clearly not advantageous, the new form reveals some other interesting aspects. First of all, equations (2.9) and (2.10) are manifestly form-invariant under gauge transformation (1.10). In particular, the electronic energy (2.7) which appears as ‘eigenvalue’ in (2.9) is gauge invariant. The third term on the left-hand side of equation (2.9), while essential for the coupling between *X* and *Φ*_*R*_, does not contribute to the energy 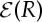, i.e. when equation (2.10) is multiplied from the left with *Φ*_*R*_(*r*)* and integrated over *r*, this term drops out. One may confirm directly that the solution of the non-adiabatic equations (2.5) and (2.6) is exact by showing that if *Φ*_*R*_(*r*) and *X*(*R*) satisfy (2.5) and (2.6), then their product will be an eigenstate of *H*_mol_
2.11
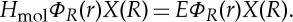


If the phase of the electronic wave function *Φ*_*R*_(*r*) depends only on *r*, and if the dependence on *R* is continuous and differentiable, then the vector potential **A**_*α*_(*R*) vanishes and (2.6) simplifies to
2.12
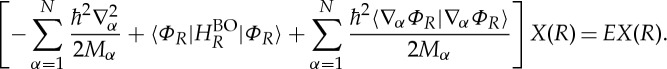


The algebraic structure of non-adiabatic nuclear equations (2.6), (2.10) or (2.12) coincides with the nuclear equation one encounters in the adiabatic approximation. However, in the latter, the electronic wave function is the BO one, satisfying
2.13
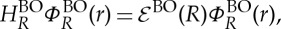
with 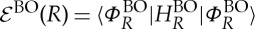, while in non-adiabatic nuclear equations (2.6), (2.10) or (2.12), the electronic wave function *Φ*_*R*_(*r*) is the non-adiabatic one satisfying (2.5) or (2.9).

The variational derivation of equation (2.6) guarantees that even in the approximate adiabatic scheme, the lowest energy eigenvalue, denoted by *E*^BO^, is rigorously an upper bound to the exact ground state energy of the system
2.14
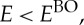
as *E* is the minimum of the molecular Hamiltonian in a Hilbert space which is wider than the Hilbert space of the adiabatic states. The inequality holds even when the electronic non-adiabatic equation is derived in a constrained way, as in the following section on the optimized effective potential (OEP) method treatment of the non-adiabatic terms. It is interesting to note that
2.15
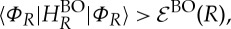
as 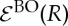 is, for each *R*, the minimum of the expectation value of 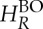.

A necessary consequence of (2.14) and (2.15) is that
2.16
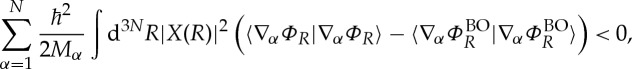
where *X*(*R*) is the lowest non-adiabatic nuclear state. Observe that all terms in the inequality are non-negative. The inequality indicates that ‘on average’ for the non-adiabatic electronic state that corresponds to the lowest nuclear state 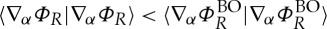. So, *Φ*_*R*_ can be expected to change less rapidly than *Φ*^BO^_*R*_ as a function of *R*. It is worthwhile to remember that when a nucleus samples a region of a degeneracy or an avoided crossing of electronic levels, the concept of the adiabatic surface loses its meaning and a visible indication of such a situation is that the potential energy depends strongly on the nuclear position. Small changes in *R* result in large changes in the wave function and potential. A lot of effort has been invested in constructing a different basis in the space of (near) degenerate electronic wave functions with energies that would behave smoothly close to the (avoided) level crossing. A convenient choice is the diabatic basis [16], defined as the basis that diagonalizes the off-diagonal matrix elements 〈*Φ*_*R*,*m*_|∇_*α*_*Φ*_*R*,*n*_〉 [16,17]. It is known, however, that rigorously such a basis does not exist in general [17,18]. The non-adiabatic electronic wave functions and energies may offer a physical solution to this problem. Within the adiabatic approximation, there are cases when the vector-like potentials **A**_*α*_ cannot all be made to vanish (for example, when adiabatic electronic levels become degenerate at a point *R*), leading to a pseudo-magnetic field in (2.6) and a geometric phase [19]. An interesting question is whether the geometric phase is a consequence of the approximate nature of the adiabatic approximation, and consequently whether it would vanish in the exact non-adiabatic scheme. The answer to this question is presently not known. Non-analyticities appearing in the 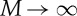 limit [20] may play a role in this context. If the geometric phase is not an artefact of the adiabatic approximation but a result of parametric decomposition (1.2) of the molecular wave function which, by itself, is not an approximation, then the framework presented here will provide a way of determining the ‘exact Berry phase’.

## 3. Approximations for the electronic non-adiabatic states

The exact non-adiabatic electronic equation is naturally very hard to solve and one has to resort to approximation techniques. A perturbative treatment of the non-adiabatic terms in the electronic equation is straightforward to apply if we keep the nuclear wave function *X*(*R*) fixed. However, when the non-adiabatic terms are large (which is the case of interest here), the expansion may converge very slowly, or not converge at all. Another method to deal with the non-adiabatic electronic equation, which seems particularly promising, is to seek for the local *R*-dependent potential *V*_*R*_(**r**) that best approximates the effect of the non-adiabatic terms in (2.5), in a manner analogous to the OEP method [21–23].

Remember that the non-adiabatic equation was derived under the constraint of normalization of electronic wave function (1.8). This was the most general way of requiring that the electronic wave function depends parametrically on the nuclear positions. A more restrictive but perhaps more physical way is to impose that the electronic wave function must be an eigenstate of a Hamiltonian which is the sum of 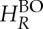 and an electronic potential *V*_*R*_ that depends on the nuclear positions. The variationally best potential is determined by making the expectation value of the molecular Hamiltonian (2.1) stationary. The optimal *V*_*R*_ will in general be an electronic *N*_e_-body potential. A further approximation, to make the problem tractable, is to restrict to (local) one-body potentials. Hence, we optimize the electronic wave function *Φ*_*R*_(*r*) under the condition that it satisfies the equation
3.1
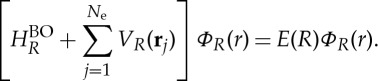
In this way, the expectation value of the molecular Hamiltonian (2.1) becomes a functional of the effective non-adiabatic potential, 〈*H*_mol_〉=*E*[*V*_*R*_].

Varying the effective potential, we obtain the functional derivative of *E*[*V*_*R*_] with respect to *V*_*R*_
3.2
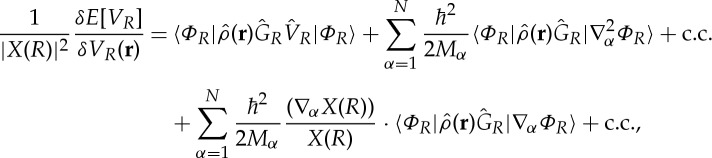
where
3.3
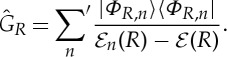
Summation is over all eigenfunctions of 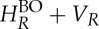, except *Φ*_*R*_. For compactness, we use in (3.2) and (3.3) second-quantization notation, 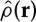 is the electron charge density operator and 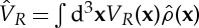. Obviously, 
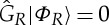
.

The OEP we seek corresponds to a stationary point of *E*[*V*_*R*_], i.e. the functional derivative vanishes
3.4
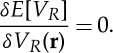


Solving the OEP equation (3.4) to obtain the potential can be very expensive. The approximation proposed by Krieger, Li and Iafrate [24,25] (KLI) can be used to simplify the equation considerably. When the electronic state is a Slater determinant of Kohn–Sham spin-orbitals 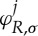, one obtains the spin-dependent KLI effective potential
3.5
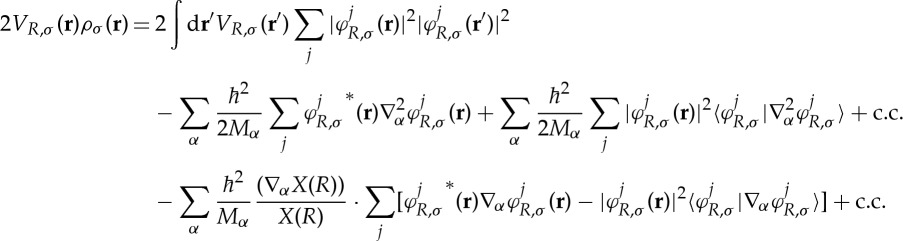
This equation can be solved within the ordinary Kohn–Sham self-consistency loop: the potential *V*_*R*,*σ*_(**r**) to be used in the next iteration is obtained by plugging the Kohn–Sham orbitals and the *V*_*R*,*σ*_(**r**) of the previous iteration in the right-hand side of equation (3.5).

We have solved the non-adiabatic OEP equation for the simplest system that contains non-adiabatic couplings, namely the hydrogen ion 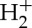. Using the BO scheme, one obtains an excellent approximation for the ground state wave function, *Ψ*(**r**,**R**)=*Φ*^BO^_**R**_(**r**)*X*^BO^(**R**). To test our method, we used the inverse BO separation, i.e. we wrote *Ψ*(**r**,**R**)=*ϕ*^iBO^_**r**_(**R**)*χ*^iBO^(**r**), where *ϕ*^iBO^_**r**_(**R**) is the conditional probability density amplitude for the relative nuclear coordinate, when the electron is frozen at *r*, and *χ*^iBO^(**r**) is the marginal probability density amplitude for the electron, moving in the potential surface of the nuclei. The inverse BO separation ignores terms of the order *M*/*m* and is hence very poor. Including the non-adiabatic correction terms (in this case, the non-adiabatic terms can be transformed to have the form of a potential, and hence the OEP method is exact), we found that the solution converged numerically to the correct wave function one obtains doing the BO separation in the orthodox way.

## 4. Summary

We have revisited the adiabatic/BO separation of the electronic and nuclear degrees of freedom in molecules, aiming to improve accuracy beyond the adiabatic level, but keeping at the same time the language and concepts of the adiabatic approximation which form the basis of almost all studies of molecular systems. We have derived a non-adiabatic correction for the electronic equation, which couples in a self-consistent way the electronic and nuclear wave functions. This correction has the form of an effective potential. The implementation of this correction to electronic structure codes that already use the OEP method to account for electronic exchange and correlation seems particularly promising.
